# Full Text Clustering and Relationship Network Analysis of Biomedical Publications

**DOI:** 10.1371/journal.pone.0108847

**Published:** 2014-09-24

**Authors:** Renchu Guan, Chen Yang, Maurizio Marchese, Yanchun Liang, Xiaohu Shi

**Affiliations:** 1 College of Computer Science and Technology, Jilin University, Changchun, China; 2 State Key Laboratory of Inorganic Synthesis and Preparative Chemistry, College of Chemistry, Jilin University, Changchun, China; 3 College of Earth Sciences, Jilin University, Changchun, China; 4 Department of Information Engineering and Computer Science, University of Trento, Trento, Italy; Shenzhen Institutes of Advanced Technology, China

## Abstract

Rapid developments in the biomedical sciences have increased the demand for automatic clustering of biomedical publications. In contrast to current approaches to text clustering, which focus exclusively on the contents of abstracts, a novel method is proposed for clustering and analysis of complete biomedical article texts. To reduce dimensionality, Cosine Coefficient is used on a sub-space of only two vectors, instead of computing the Euclidean distance within the space of all vectors. Then a strategy and algorithm is introduced for Semi-supervised Affinity Propagation (SSAP) to improve analysis efficiency, using biomedical journal names as an evaluation background. Experimental results show that by avoiding high-dimensional sparse matrix computations, SSAP outperforms conventional k-means methods and improves upon the standard Affinity Propagation algorithm. In constructing a directed relationship network and distribution matrix for the clustering results, it can be noted that overlaps in scope and interests among BioMed publications can be easily identified, providing a valuable analytical tool for editors, authors and readers.

## Introduction

With the proliferation of biomedical research and publications, individual scientists can no longer keep track of relevant articles through reading alone. Instead, they must rely on text mining tools to explore the implicit knowledge and hypotheses presented in biomedical texts and to extract the data and concepts relevant to their work [1], [2]. In this regard, a number of new challenges have emerged, especially in relation to full text analysis [3], complex relation extraction [4], [5], and information fusion [6].

To date, research approaches to biomedical text mining have been based exclusively on article abstracts (Iliopoulos et al., 2001 [7]; Yu and Lee 2006 [8]; Zhu et al., 2009 [9]), culminating most recently in 2011, when Boyack et al managed to cluster about two million MEDLINE abstracts [10]. While such clustering can provide hints about primary results and conclusions, the full texts of articles are where more detailed methodologies, experimental results, critical discussions and interpretations are found.

Rodriguez-Esteban pointed out that full article texts are the only source for certain crucial information, such as experimental measurements [11]. Recent research initiatives in bioinformatics, such as TREC Genomics (http://ir.ohsu.edu/ge-nomics/) and BioCreAtIvE (http://www.biocreative.org/), also recommend migrating text analysis from abstracts to full texts [12]. Intuitively, consultation only of abstracts is inadequate for judicious analysis of biomedical articles, and can even lead to inappropriate clinical decisions [13]–[15].

In the Genomics2005 data collected in [9], the mean number of documents in the 100 datasets was 690.7 and the average number of unique words was 2214.4. By contrast, in our experiments using a BioMed dataset containing 600 texts, the unique word count was 54367—approximately 24 times greater than the count for Genomics2005. With the addition of these terms, it is expected that biomedical text mining will provide more relevant information and more complete analysis.

Unfortunately, access to the full text and citations of biomedical papers remains limited, and the complexity of full text mining, which must contend with large amounts of information noise, is much greater than that of abstract text mining. While the number of non-redundant words (terms) in a biomedical abstract text is typically less than 100, that of a full text is often much greater than 1000. Since most of mining frameworks employ the vector space model (VSM) [16], which treats a document as a bag of words and uses plain language terms as features, the dimensions of a full text corpus can be several times greater than that of abstract data.

Clustering is one of the most popular techniques for data analysis in many disciplines [17]. The basic strength of text clustering is its capacity to automatically organize texts into meaningful groups. As such, it has been applied in a number of ways, including cluster-based retrieval [18], key sentence extraction [19], concept discovery in molecular biology [7], and so on. Traditional clustering also forms an important component of unsupervised learning algorithms, which along with various supervised techniques, play an important role in biomedical research, including studies of cancer [20], Bayesian analysis of HIV drug resistance [21], linear regression frameworks for motif finding [22], etc.

In supervised text mining algorithms, a training process is first performed on a large set of known predefined topics and text labels. This generally results in better topic detection [23] than unsupervised approaches, for which there is no prior training step. Obviously, supervised and unsupervised learning algorithms can work together to improve learning processes or handle more complex problems, such as phosphorylation site prediction [24]. However, training set labeling is in general very costly and frequently unavailable in practical scenarios. In recent years, semi-supervised clustering approaches have caught the attention of the machine learning community. These approaches make use of a smaller and more easily obtained set of labeled samples to guide clustering strategy. They have been applied in many application domains, including text clustering [25], gene expression analysis [26], and image processing [27]. However, to the best of our knowledge, semi-supervised learning has not yet been applied in full text mining of real biomedical publications.

In this paper, the Semi-supervised Affinity Propagation (SSAP) method of text clustering is proposed. Then this method is applied to the corpus of a real biomedical text database called BioMed Central open access full-text corpus, and compare its clustering performance to that of two classical clustering algorithms. Finally, a directed relationship network and a cluster distribution matrix are constructed based on the SSAP clustering results, and use these to reveal publication interests among the top 10 journals. The source code and datasets used in this paper are available in the Supporting Information.

## Materials and Methods

### Datasets

The dataset used in the experiments was the BioMed Central open-access corpus (BioMed), which can be downloaded at http://www.biomedcentral.com/about/datamining. From the BioMed corpus, 110,369 articles between Jan. 2012 to Jun. 2012 are downloaded. Each BioMed file is a well-formatted XML document, and contains various tags, such as <title>, <bdy>, <issn>, and so on. But there are many articles contain only XML tags and abstracts, so those files of less than 4KB are removed in the pre-processing phase. Then the remaining files are divided into different ‘topics’ according to their journal names and the title, abstract and plain texts are extracted. Finally, the top 10 topics with paper numbers from 1966 to 5022 are selected as the test corpus. The detailed information of these 10 topics are listed in Table 1. In our experiments, to evaluate the performance of the different approaches across different dataset sizes, we randomly select 5 sub-datasets with scales of 400, 500, 600, 700 and 800 on each topic for comparison. And for each scale we randomly select 5 times computation to perform average to avoid accidental results.

**Table 1:** Top 10 topics in Biomed corpus.

Serial Number	Name	Abb Name	DocumentNumber
1	BMC Bioinformatics	BMC Bioinformatics	5022
2	BMC Genomics	BMC Genomics	4121
3	BMC Public Health	BMC Public Health	3758
4	BMC Cancer	BMC Cancer	3025
5	Journal of Cardiovascular Magnetic Resonance	J Cardiovas Magn R	2538
6	Retrovirology	Retrovirology	2478
7	BMC Neuroscience	BMC Neuroscience	2454
8	BMC Evolutionary Biology	BMC Evo Biol	1973
9	Malaria Journal	Malaria J	1968
10	Journal of Medical Case Reports	J Med Case Rep	1966

Before beginning the clustering process, all journal-name-related information were eliminated from target texts. After clustering, this information was used to evaluate the clustering performance of each algorithm. According to the list in the website: http://norm.al/2009/04/14/list-of-english-stop-words/, the stop words are removed. Then each document is represented by a set of tow-tuples and the whole dataset should have the form indicated as follows:
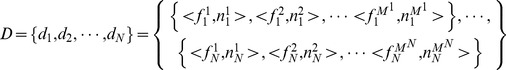
where *N* is the document number in the dataset, *M^j^* is the term feature (unique words or phrases) number in the *j*th document, *d_i_* = {<*f_i_* ^1^, *n_i_* ^1^>, <*f_i_* ^2^, *n_i_* ^2^>,…<*f_i_^M^* ^i^, *n_i_^M^* ^i^>} indicates the *i*th document, and *f_i_* ^k^ and *n_i_* ^k^ represent the *k*th term in *i*th document and its normalized frequency, respectively. Hereinto, the normalized frequency is computed by
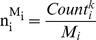
where *Count^k^_i_* is the *k*th term count number in the *j*th document.

### The Semi-Supervised Affinity Propagation Method

Based on the state-of-the-art unsupervised Affinity Propagation clustering algorithm [19], SSAP method is proposed as means of addressing the complexity problems posed by full text clustering of biomedical publications. In conventional vector space model (VSM) based methods, each document is represented in the feature space constructed by all the unique word or phrase of all the documents. Therefore, the similarities between different document pairs could be computed according to any similarity measurements under the same constructed space. However, this space is high-dimensional and the document representation is serious sparse. To avoid the large dimensions and sparse matrix computation, in the proposed method each document is represented in a tiny sub-space of VSM when computing the similarity between it and another one. The sub-space is spanned by only the features of the related document and its counterpart, which is much smaller than the original vector space. If the dataset includes thousands of words or phrases, the subspace restriction will reduce computational complexity significantly. In the method, the classical similarity measurement in text clustering, namely, Cosine Coefficient similarity is used. To achieve even better performance, a semi-supervision strategy that makes use of known information is introduced. The detailed steps of the SSAP method are as follows:

*a*. *Initialization of dataset*: Initializing dataset *D* = {*d*_1_, *d*_2_,...,*d_N_*} be a superset of *N* (*N*>0) elements, where each element consists of a sequence of two-tuples;

*b*. *Seed Construction*: Add a small number of initially labeled objects in two-tuple sets to the dataset *D*, and get a new dataset *D′* containing *N′* elements (*N′*≥*N*).

*c*. *Similarity Computation*: Compute the similarities among objects in *D′* using the Cosine Coefficient similarity metric:
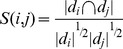
where *S*(*i*,*j*) is the similarity between the *i*th document and the *j*th document elements in *D*′, and |*d*| represents the unique terms' count number in document *d*, respectively.

*d*. *Self-Similarity Computation*: Compute the self- similarities *s(l, l)* of each object in *D′* using: 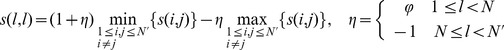
where 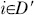, 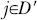, and *i*≠*j*.

*e*. Initialization of availability matrix: *a*(*i*, *j*) = 0, (*i*, *j*  = 1, 2,⋯..., *N*′).

*f. Message Matrix Computation:* Compute the message matrices using:
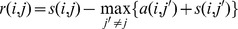


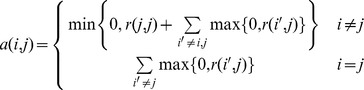



*g*. *Exemplar Selection*: Add the two message matrices and search for the exemplar of each object *i*, defined as the maximizer of *r*(*i*, *j*)+*a*(*i*, *j*).

*h. Updating Messages*, using:
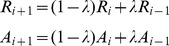



*i*. *Iterating from Step f to h* for a fixed number of iterations or until the exemplar selection remains constant for some number of iterations.

*j. Merging Small Clusters* with the same labels into larger clusters to produce clustering output.

### Comparison Methods

SSAP is proposed based on Affinity Propagation (AP) method, which was proposed by Frey and Dueck in *Science* in 2007 [19] and was considered a convinced clustering algorithm in different applications. To investigate the semi-supervised learning performance of SSAP, AP is set as a comparison method in the experiments. K-means was firstly proposed by MacQueen [28] and was recognized as one of the top 10 algorithms in data mining [29]. Especially, it has proven that K-means is a simple but very reliable method in document clustering [30], [31]. Therefore, it is also used as a baseline for comparison.

### Evaluation

To evaluate the performance of the three clustering methods—k-means, Affinity Propagation and SSAP, their respective values for F-measure, entropy, and CPU execution time are compared. For information retrieval, entity recognition, and information extraction in particular, F-measure is the most commonly used evaluation measurement. The global F-measure for the entire clustering result is defined as:
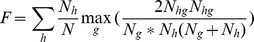
where *N* is the total number of documents in the dataset, *N_hg_* is the number of objects of class *h* in cluster *g*, *N_g_* is the number of objects of cluster *g*, and *N_h_* is the number of objects of class *h*.

In contrast to F-measure, entropy provides a measure of the homogeneity or purity of a cluster. The total entropy for a set of clusters is calculated as:
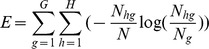
where *G* is the total number of clusters, *H* is the number of predefined classes. The smaller the entropy, the better the clustering performance.

The generated clusters with the set of journal-based topic categories in BioMed are examined. To check the effectiveness across different collection sizes, all the three algorithms are applied to the sub-datasets with 5 scales from 400 to 800 and for each scale the sub-datasets are randomly selected 5 times for averaging. All topics in the datasets had discrete uniform distributions, and all experiments were run on a PC equipped with Intel (R) Xeon(R) CPU×3430 @ 2.40 GHz, 2 GB of Ram, and no parallel computing processes.

## Results

**Figure 1:**
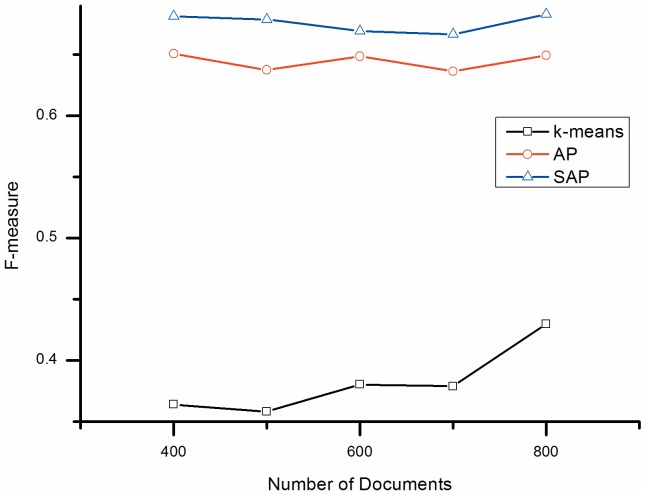
F-measure comparison. K-means: k-means clustering; AP: Affinity Propagation clustering; SSAP: Semi-supervised Affinity Propagation.

**Figure 2:**
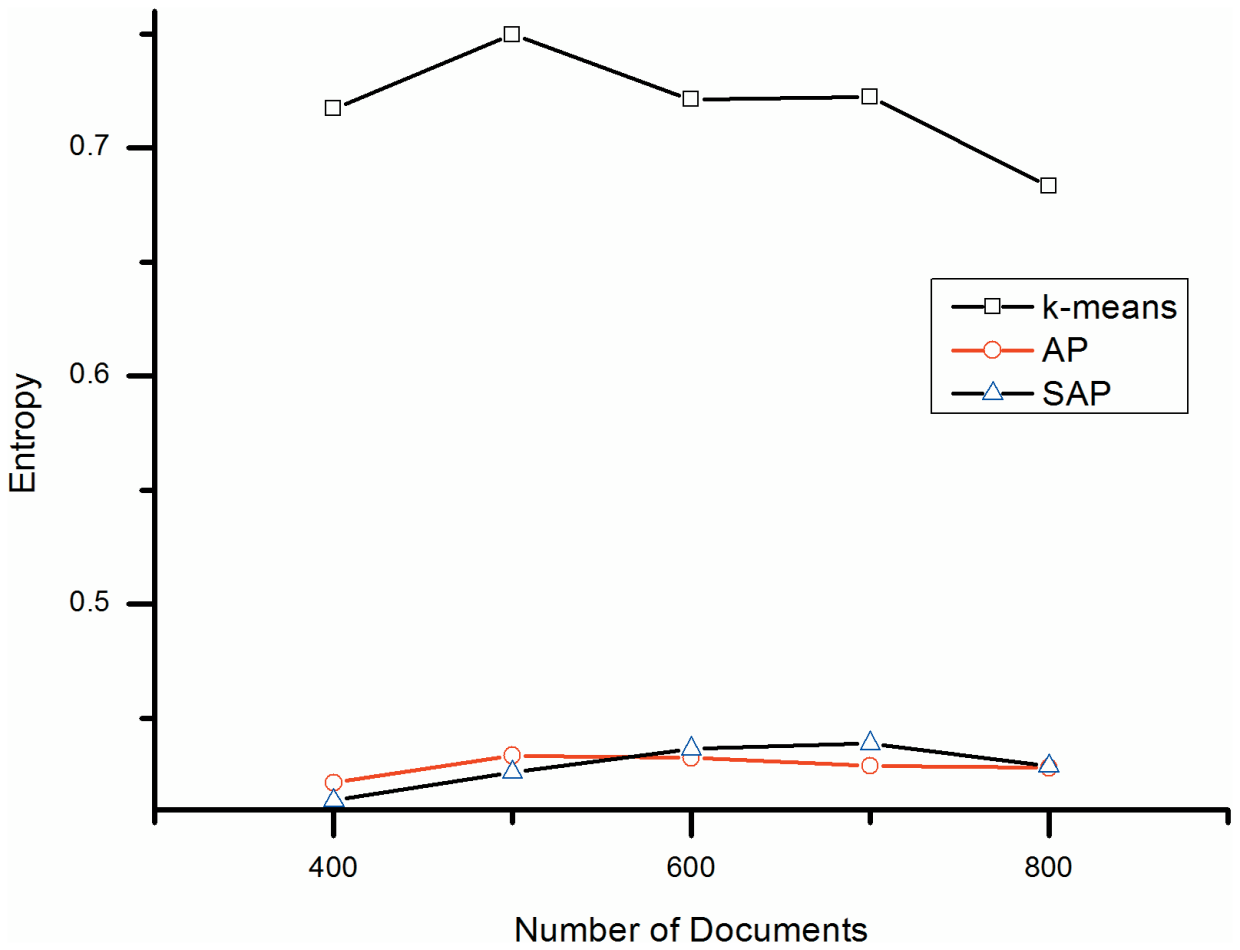
Entropy comparison. K-means: k-means clustering; AP: Affinity Propagation clustering; SSAP: Semi-supervised Affinity Propagation.

**Figure 3:**
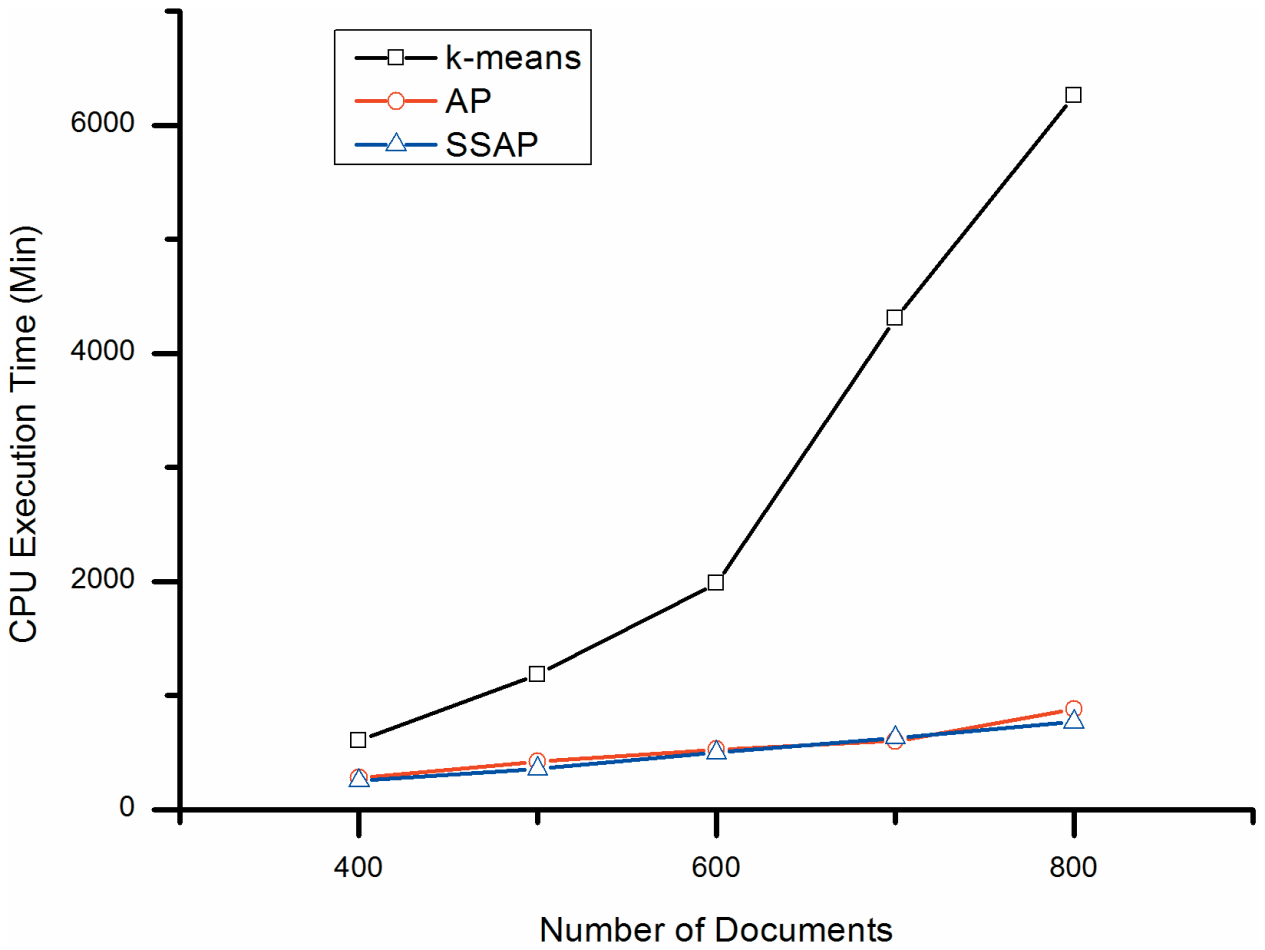
CPU execution time comparison. K-means: k-means clustering; AP: Affinity Propagation clustering; SSAP: Semi-supervised Affinity Propagation.

Figures 1, 2, and 3 compare the F-measure, entropy and CPU execution time measurements, respectively, for the three algorithms. From Figure 1 and the summary results in Table 2, it can be seen that the mean F-measure value of SSAP is 0.674, an improvement of 0.290 (75.4%) over k-means. Similarly, AP achieves a mean F-measure of 0.650, an improvement of 0.266 (69.1%) over k-means.

**Table 2:** The mean values over all experiments.

	Mean F-measure	Mean Entropy	Mean CPU execution time (Min)
SSAP	**0.674**	**0.429**	**498.9**
AP	0.650	**0.429**	539.6
k-means	0.384	0.721	2866.8

Figure 2 and Table 2 show a different trend for the entropy values of the three methods, with k-means showing the highest entropy and both SSAP and AP achieving around 40.0% lower entropy than k-means.

From Figure 3, it is clear that the CPU execution times for AP and SSAP are much lower than that of k-means, for equivalent performance results. Moreover, the gaps enlarge exponentially as the dataset size increases. For example, k-means took 2.4 times longer to execute than SSAP for the 400-document dataset, and increased to 8.2 times for the 800-document dataset. This result agrees with the conclusions of other studies, such as Frey and Dueck's papers in *Science* [19], [32]. Furthermore, even after 10000 runs of 400-document clustering, the k-means could only achieve an F-measure of 0.384 and entropy of 0.721, well below the performance of SSAP. To obtain the best result, k-means needs to execute all possible solutions, the equivalent of about *C^10^*_400_≈2.57×10^19^ runs for 400 documents [33].

In addition, the CPU run time comparison results shown in Figure 3 and the summary results in Table 2 indicate that SSAP is about 7.5% faster than AP. This is due to the fact that, with the added labeled texts and semi-supervised strategy, the unlabeled texts are more easily to find their clusters, the convergence of AP are more quickly. Moreover, SSAP performs better (by 3.8%) than AP with respect to F-measure and similar to AP in average entropy.

For the 5data sizes (400, 500, 600, 700 and 800 texts), the adjustable factors *φ* were, respectively, 0.5∼1 for AP, and 3 for SSAP. The parameter *k* in the k-means execution for all experiments is set as 10 because the documents are expected to be clustered into 10 classes corresponding to the top 10 journals. It should be noticed that there are some methods have been developed to select or construct seeds in semi-supervised methods, which could improve the performance when we have complicated known knowledge. However, the feed construction strategy is not the focus of this paper and there are no discusses about this. In the experiments, we just simply randomly select 4 documents in each cluster to guide the seed construction in SSAP algorithm.

### Relationship Network of Publications

**Figure 4:**
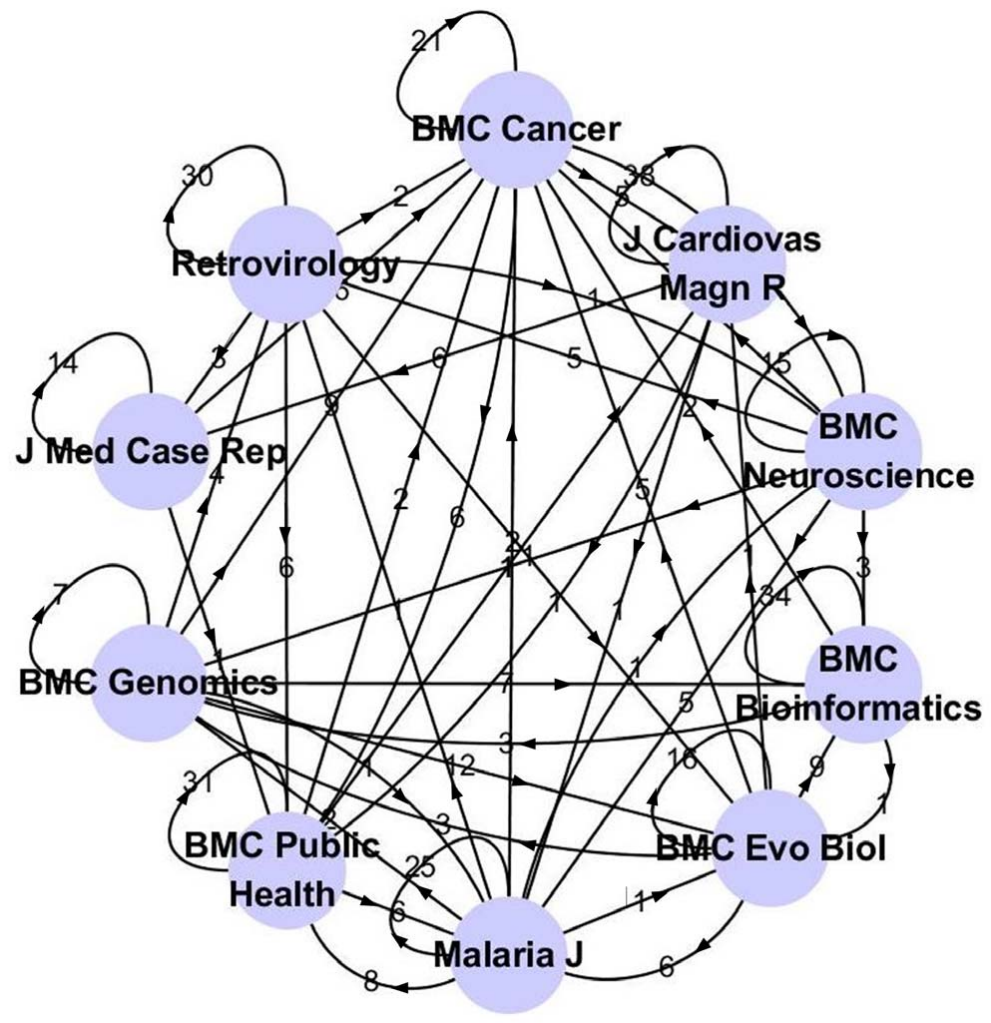
Directed relationship network based on SSAP clustering of BioMed journals. Each node indicates a biomedical journal.

Based on the SSAP clustering results, an interesting application of our method is demonstrated. Figure 4 describes the relationships among the 10 top biomedical journals yielding a total of 400 articles. Within the graph, each node represents a biomedical journal. If a directed edge from journal A to B exits, it shows that at least two articles of journal A are clustered into the class dominated by journal B, and the weight of the edge represents the count number of the articles.

In Figure 4, it can be seen that most of these journals share topics with other journals. A detailed analysis of the graph shows that “BMC Cancer” gets eight out-degrees, which means that it has outward connections with most of the other journals except for the “Journal of Cardiovascular Magnetic Resonance”. By contrast, the “Journal of Medical Case Reports” has no out-degrees. The median out-degrees of the 10 journals in the network is 3. “BMC Bioinformatics”, “BMC Public Health”, “Malaria Journal” and "Retrovirology” all belong to this group. The out-degrees of “BMC Genomics” and “Journal of Cardiovascular Magnetic Resonance” are 4 and 2, respectively.

Table 3 gives the detailed parameter information of the relationship network. It can be seen that the clustering coefficient is 0.36 which is larger than 0, as most random world networks tend to be, but also much smaller than 1, which is a typical feature of ring-lattice world networks. It can also be noted that the characteristic path length is 1.89, which means that these nodes are well connected. Based on these two parameters, it can be concluded that the relationship network is similar to that of a small world [34].

**Table 3:** Parameter analysis of the directed relationship network for SSAP clustering of biomed journals.

Number of nodes:10	Number of inter edges:27
**Clustering coefficient:0.36**	Connected components:1
Network radius:2	Network diameter:5
Shortest paths:73	Network density: 0
**Characteristic path length:1.89**	Avg.number of neighbors:4.4

Table 4 is the result of cluster distribution matrix. The principal diagonal of the table indicates the match number of the clustering results and the journal name of BioMed corpus. They are also the main parts of their clusters. The homologous texts are those belonging to other journals but are recognized under the given cluster label. For example, in cluster 1, there are 19 homologous texts, including nine articles from “BMC Evolutionary Biology”, seven from “BMC Genomics”, and three from “BMC Neuroscience”.

**Table 4:** Cluster distribution matrix.

Cluster	1	2	3	4	5	6	7	8	9	10	Amount
BMC Bioinformatics	**34**	1	3	0	2	0	0	0	0	0	40
BMC Evo Biol	9	**16**	3	0	5	0	6	1	0	0	40
BMC Genomics	7	12	**7**	0	9	0	1	0	4	0	40
BMC Neuroscience	3	0	1	**15**	*11*	0	5	0	5	0	40
BMC Cancer	0	0	0	*8*	**21**	6	0	5	0	0	40
BMC Public Health	0	0	0	0	2	**31**	*6*	1	0	0	40
Malaria J	0	1	2	1	2	*8*	**25**	0	1	0	40
J Cardiovas Magn R	0	0	0	0	0	1	1	**38**	0	0	40
Retrovirology	0	1	0	1	2	6	0	0	**30**	0	40
J Med Case Rep	0	0	0	0	16	1	0	6	3	**14**	40
Amount	53	31	16	25	70	53	44	51	43	14	400
Homologous texts	**19**	**15**	**9**	**10**	**49**	**22**	**19**	**13**	**13**	**0**	**169**

## Discussions

### Discussions on Full Text Clustering Results

From the experimental results on full text clustering experiments, it can be seen that SSAP is superior to the classical k-means and AP algorithms for full text biomedical literature clustering. With the help of local similarity computing and semi-supervision, SSAP greatly enhances the clustering performance relative to k-means. Applying AP clustering to replace k-means clustering, the two AP-based algorithms obtained higher F-measures and lower entropies. This is due to the fact that the Cosine Coefficient similarity contains both the document's own information and a portion of the mutual information for the two vectors which would have been omitted by Euclidian distance. By introducing the semi-supervised strategy, the SSAP algorithm outperforms the unsupervised AP algorithm, achieving a higher F-measure than AP while maintaining similar computation times. In addition, because the k-means method treats each document as a 54367-dimension vector (i.e. 54367 unique words in 600 texts) using Euclidian distance, the problem is mapped into a large sparse matrix, which dramatically increases the computation time. By contrast, SSAP and AP treat each document as a much smaller vector space (i.e. at most contains 5274-dimension), which allows them to execute much more quickly.

### Discussions on Relationship Network Analysis

In relationship network analysis, it is assumed that the manuscripts in the same journal share similar topics and have high possibility to be clustered in a same class. However, similar papers may also exist in different journals. Since the selected dataset belongs to a discrete uniform distribution, the edge between two journals in the relationship network can reveal similarity of their publishing scopes and strategies. For example, the scope of “BMC Bioinformatics” is mostly defined by “computational and statistical methods for the modeling and analysis of all kinds of biological data” [35], and it can be seen that the journal has strong relationships with most other BMC series journals, including out-degrees for “BMC Genomics”, “BMC Neuroscience”, and “BMC Evolutionary Biology”, as well as an in-degree with “BMC Cancer”. This is due to the fact that these journals belong to the same publishing company and “BMC Bioinformatics” defines a broad publishing scope, including all computing related models for all kinds of biological data. Moreover, with cancer research attracting so much attention and covering such diverse biomedical topics, the papers published by “BMC Cancer” are featured in many different research areas, including tissue analysis, diagnosis, and treatment of tumors. This explains why the “BMC Cancer” journal forms a central hub connected to most of the other journals. By contrast, the “Journal of Cardiovascular Magnetic Resonance” and “Journal of Medical Case Reports” are more focused on specific fields (e.g., “magnetic resonance methods applied to the cardiovascular system” [36], or “case report that expands the field of general medical knowledge, and original research relating to case reports” [37]). As a result, these journals have fewer in-degrees and fewer out-degrees.

The cluster distribution matrix is an intuitive and informative tool for analysis, from it, it can be noted that the following:

1. **Search area or publishing strategy relationships.** “BMC Cancer” has the most homologous texts (49 texts), while “Journal of Medical Case Reports” has the fewest (0 texts). This may be due to the latter's relatively narrow research area (medical case study), which excludes the majority of biology manuscripts. On the other hand, it indicates that the editors and reviewers of this journal are more concentrated on particular medical cases.
2. **Publishing scope relationships.** It is clear that the wider the publishing scope, the more homologous texts can be found. For example, the number of homologous texts for “BMC Bioinformatics” is greater than that of “BMC Evolutionary Biology,” which is in turn greater than that of **“**BMC Genomics”. This finding fits with the scope of these journals' research areas: “BMC Bioinformatics” focuses on “the development, testing, and novel application of computational and statistical methods for the modeling and analysis of all kinds of biological data, as well as other areas of computational biology” [35]; “BMC Evolutionary Biology” focuses on “molecular and nonmolecular evolution of all organisms, as well as phylogenetics and palaeontology” [38]; and **“**BMC Genomics” focuses on “genome-scale analysis, functional genomics, and proteomics” [39]. It is obvious that a publishing scope encompassing “all kinds of biological data, as well as other areas of computational biology” is larger than that encompassing just “molecular and nonmolecular evolution” or “genome-scale analysis”.
3. **Mutual cross-relationships.** Mutual cross-relationship between two journals includes the weights of both directions between these two nodes in the journal relationship network (Figure 4), or equivalently, the two elements crossed by these two journals in the cluster distribution matrix (Table 4). It can reflect the publishing scope similarity between the related two journals. It could be found that the mutual cross-relationships between “BMC Public Health” and “Malaria Journal” and those between “BMC Neuroscience” and “BMC Cancer” are significantly bigger than others (see the numbers indicated by the italicized and underlined in Table 4), which means that the journals have homologous texts in the given partner's cluster, and suggests that the publishing scopes of the two journals have some overlap. For example, it is well known that malaria is one of the epidemics which threatens human's public health. So it is easy to understand “BMC Public Health” and “Malaria Journal” include part of similar papers. For the second pair journals, we know that cancers of the brain and nervous system (a subcategory of cancer) are the second most common type of childhood cancer, which explains why the mutual cross-relationship between “BMC Neuroscience” and “BMC Cancer” is the most significant one among all the journals.

## Conclusions

In this paper, we addressed the difficulties of full text clustering of biomedical publications by proposing a new method and algorithm known as SSAP. To reduce the huge dimensionality when the size of targeted texts is increasing, the new method substitutes pair-wise vector-spanned sub-space for the entire Euclidean space which are used by classic algorithms. To solve the nonmetric similarities problem, the proposed algorithm employs Affinity Propagation clustering, the performance of which is further improved by a small sampling of labels. Using the real-world corpus of BioMed Central as a target dataset, the performance of SSAP is compared to that of two classical clustering algorithms, and it can be seen that: (1) SSAP clustering thoroughly outperforms k-means clustering, and (2) SSAP clustering improves upon unsupervised AP clustering, with minimal impact on computation time.

The study of the directed relationship network and distribution matrix based on SSAP clustering results also demonstrated the utility and applicability of the proposed method within the biomedical field. In particular, or identification of publishing scope and mutual cross- relationships among journals provided a case study of how a powerful clustering algorithm can be used to detect meaningful links among various scientific journals.

## Supporting Information

Dataset S1 **Sub-datasets with the scale of 400.** Each journal contains 40 manuscripts.
(RAR)

Dataset S2 **Sub-datasets with the scale of 500.** Each journal contains 50 manuscripts.
(RAR)

Dataset S3 **Sub-datasets with the scale of 600.** Each journal contains 60 manuscripts.
(RAR)

Dataset S4 **Sub-datasets with the scale of 700.** Each journal contains 70 manuscripts.
(RAR)

Dataset S5 **Sub-datasets with the scale of 800.** Each journal contains 80 manuscripts.
(RAR)

Source Code S1 **Source code of SSAP algorithm.**
(HPP)
